# 996. CD4+ T-Cell Lymphopenia Associated with Frequent Plateletpheresis in Healthy Donors

**DOI:** 10.1093/ofid/ofab466.1190

**Published:** 2021-12-04

**Authors:** Phoebe H Cunningham, Xhoi Mitre, Djenane Pierre, Christina Montesano, Tenaizus Woods, Karina Oganezova, Jonathan H Krauss, Salena S Von, John A Kupelian, Jon A Gothing, Kleinjan Jane, Lise Ann Caldara, Amy C Sherman, Stephen R Walsh, Richard M Kaufman, Lindsey R Baden, Michaël Desjardins

**Affiliations:** 1 Brigham & Women’s Hospital, Brookline, Massachusetts; 2 Brigham and Women’s Hospital, Boston, Massachusetts; 3 Brigham & Women’s Hospital, Boston, Massachusetts; 4 Brigham and Women’s, Watertown, Massachusetts; 5 Brigham & Womens Hospital, Boston, Massachusetts; 6 Harvard Medical School/Brigham and Women’s Hospital, Jamaica Plain, Massachusetts

## Abstract

**Background:**

Frequent plateletpheresis using the Time Accel leukoreduction system chamber may result in lymphopenia in healthy donors, with increased donation in the previous year associated with CD4+ T-cell count of less than 200 cells/µL. However, this finding has not been replicated and the clinical significance of plateletpheresis-associated lymphopenia remains unclear.

**Methods:**

A prospective observational study of healthy plateletpheresis donors aged 18 or older who donated at least once in the previous 365 days was conducted at the Kraft Blood Center at Brigham and Women’s Hospital/Dana Farber Cancer Institute, where the Time Accel system is used exclusively. Blood was drawn immediately before plateletpheresis or at least 2 weeks after the last donation to assess for total lymphocyte and CD4+ T-cell counts.

**Results:**

A total of 86 participants were enrolled: 23 had 1-5 donations, 36 had 6-19 donations, and 27 had 20-24 donations within the previous 365 days (Figure 1). For the low-, medium-, and high-frequency donation groups, the median age was 53 years (IQR 43-64), 61 years (IQR 53-68), and 61 years (IQR 55-65), respectively. The median total lymphocyte count was 1.5 (IQR 1.3-1.9), 1.2 (IQR 0.9-1.5), 0.8 (IQR 0.6-0.9) 10^3^ cells/µL, and the median CD4+ T-cell count was 648 (IQR 531-843), 525 (IQR 348-698), and 220 (IQR 184-347) cells/µL. CD4+ T-cell counts were < 200 cells/µL in 0/23 (0%), 3/36 (8%), and 9/27 (33%) participants across the three groups. Total lymphocyte and CD4+ T-cell counts were inversely correlated with the number of platelet donations in the prior 365 days, R^2^ = 0.384 (Fig 2) and 0.402 (Fig 3) respectively.

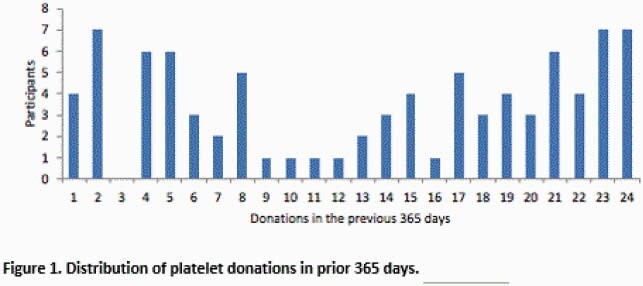

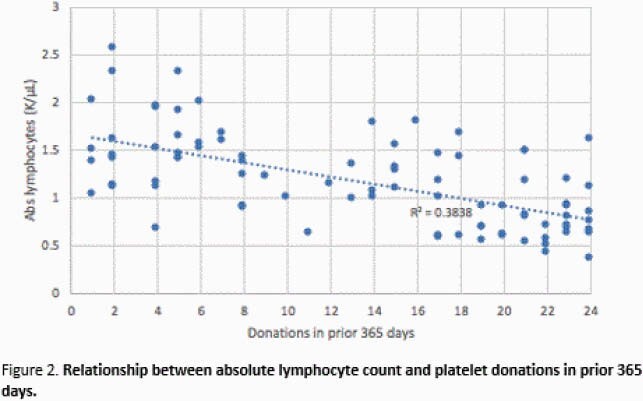

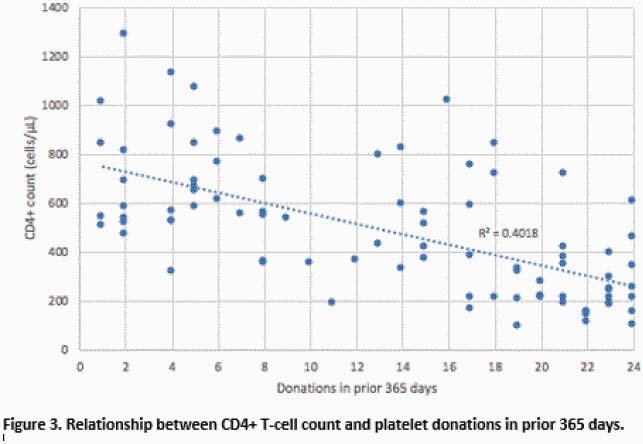

**Conclusion:**

Frequent plateletpheresis using Time Accel leukoreduction system chamber is associated with CD4+ T-cell lymphopenia, with counts below 200 cells/µL seen in one third of those who donated 20-24 times in the previous year. Vaccine immunogenicity studies are ongoing to evaluate the clinical significance of this finding.

**Disclosures:**

**Stephen R. Walsh, MDCM**, **Janssen Vaccines** (Scientific Research Study Investigator)**Regeneron** (Scientific Research Study Investigator)**Sanofi Pasteur** (Scientific Research Study Investigator)

